# Meaning-Making Through Dialogic Classroom Discourse in History Classes: Multi-Perspective Case Studies From a Teacher Professional Development Program

**DOI:** 10.1177/23522798241278284

**Published:** 2024-09-11

**Authors:** Miriam Moser, Matthias Zimmermann

**Affiliations:** 1 University of Freiburg, Switzerland; 2 University of Teacher Education St. Gallen, Switzerland

**Keywords:** dialogic teaching, professional development, student participation, video research, classroom discourse

## Abstract

This article examines the characteristics of meaning-making during classroom discourse using data from a study regarding a yearlong teacher professional development (TPD) program intended to promote dialogic discourse in whole-class practice. The in-depth, video-based case analyses of two whole-class discussions in history classes (two classes/teachers, *N* = 46 students) at the end of the TPD integrate multi-semiotic and content-bound perspectives. The analyses show how the students adopt an active role in shaping the dialog and contribute to the direction of the discourse, while both teachers assume a moderating stance. Concurrently, the findings highlight the inherent tension teachers face while ensuring student participation and compliance with disciplinary norms. In addition, the results reveal new student roles and asymmetries due to altered responsibilities in student-owned classroom discourse, which are particularly elucidated through nonverbal interactions. This contributes to existing research by highlighting novel, critical elements for consideration in TPD.

## Introduction

In classrooms around the world, teachers dedicate most of their teaching time to interacting with the entire class (Alexander, 2008, 2020; Hiebert et al., 2003; Pauli, 2010; cf. Schnitzler et al., 2021). Based on Vygotsky’s (1978) sociocultural theory, such whole-class interactions play a crucial role in the learning process, particularly when students are engaged as equal co-constructors of meaning. A growing body of empirical research confirms the potential of such dialogic classroom discussions that provide students with opportunities to articulate and refine their ideas with their peers (cf. Alexander, 2020; Hattie, 2023; Pauli & Reusser, 2018; Resnick et al., 2015, 2018a; Teo, 2019; Walshaw & Anthony, 2008). Despite the prevailing consensus regarding the educational value of dialogic classroom discourse, empirical research indicates that dialogic discourse remains the exception, rather than the rule, in everyday classrooms (Alexander, 2015; Osborne et al., 2019; Resnick et al., 2018a). Instead of leading a discussion that invites student participation, teachers often use simple recall questions that provide students with little opportunity to engage in meaningful discourse (e.g., Resnick et al., 2018a; Schuitema et al., 2017). In such a traditional lecture-like classroom discourse, teachers assume the role of discussion leader and meaning owner, while students serve as keyword givers (Kobarg & Seidel, 2007; Pauli, 2006; Walsh, 2006).

To address this issue and promote dialogic classroom discourse, a number of approaches have been developed (e.g., Dialogic Teaching and Academically Productive Talk; cf. Kim & Wilkinson, 2019) and implemented in the context of teacher professional development (TPD). In our study Socrates 2.0, we aimed to support teachers in fostering dialogic classroom discourse in their teaching practice through a TPD program based on the Accountable Talk approach (Michaels et al., 2008). Our prior findings (Moser et al., 2022; Zimmermann, 2023) align with those of numerous other studies (Boyd, 2023; Chen et al., 2020; Pehmer et al., 2015; Scott et al., 2006) demonstrating the positive effects of dialogic classroom discourse on teacher and student communication using frequency analyses, such as analyses of talk share and interaction patterns.

Nevertheless, previous analyses, including those conducted by the authors in 2022 and 2023, have not yet considered the content-related process of meaning-making in dialogic classroom discussions. Moreover, there is a paucity of research analyzing classroom discourse by examining both the verbal and multimodal dimensions of teacher-student interaction (Taylor, 2014). This article is intended to address this gap through an in-depth case analysis of two classroom discussions during two history classes conducted as part of a TPD program. The objective is to unpack the emergence of meaning by closely examining how meaning and the relevance of the subject matter are negotiated by the students and their teacher. The focus is primarily on the interplay between student and teacher actions, rather than individual statements. Additionally, this study exploits the multimodal dimension of communication (e.g., Babad, 2008) as a critical component, alongside verbal language, in the examination of teacher-student interactions.

## Theoretical Framework

The following section begins by outlining the relevance of classroom discourse for students’ meaning-making in history class. Subsequently, the overall challenges involved in implementing dialogic class discussions are illustrated. We then provide an overview of the present research status regarding recent TPD programs fostering dialogic classroom discussions. Lastly, the concept of Accountable Talk, which underpins the TPD framework utilized in our study, will be elaborated.

### The Relevance of Classroom Discourse for Meaning-Making in History Class

According to Bruner (1990), meaning is “created and negotiated within a community” (p. 11). Therefore, from a generic perspective, meaning is taken to be a product of social processes, particularly social interactions. In line with Vygotsky’s (1978) sociocultural theory, learning is always connected with processing information articulated during social interactions. As stated by Zittoun and Brinkmann (2012), “Learning as meaning-making” emphasizes “the fact that in any situation of learning, people are actively engaged in making sense of the situation—the frame, objects, relationships—drawing on their history of similar situations and on available cultural resources” (p. 1809). To make sense of a social situation, people interact with one another and open a dialog to resolve socio-cognitive conflicts (Bakhtin, 1981). Littleton and Mercer (2013) describe dialogic meaning-making as *interthinking*, illustrating how “people can combine their intellectual resources to achieve more through working together than any individual could do on their own” (p. 111). In a classroom context, dialogic whole-class discussions offer particular potential in terms of learning. Despite asymmetrical levels of competence (teacher as expert, students as apprentices), the class and the teacher form a community of learning, which develops knowledge in a co-constructive manner and negotiates meaning together (e.g., Alexander, 2020; Michaels et al., 2010). Interactional norms of dialogic classroom talk position students as authors of ideas who are prompted to make substantial content-based contributions to the discussion (O’Connor & Michaels, 1996). In this form of social interaction, the student “acquires a framework for interpreting experience and learns how to negotiate meaning in a manner congruent with the requirements of the culture” (Bruner & Haste, 2010, p. 1).

Given that history is a fundamental element of every culture, it is essential for students to make meaning from historical phenomena (Knudsen, 2020; Popa, 2022). To support learning in history, the teacher’s primary role, according to Halldén (1994), is to frame history teaching as an ongoing discourse in which a shared line of reasoning is established through the constant collaborative negotiation of meaning based on historical artifacts. Students already possess certain dispositions that are necessary to engage with history based on everyday meaning-making and grappling with temporalities (Bruner, 2005; summary in Zimmermann, 2023). However, because every historical learning object evokes different objective and subjective presuppositions, it is difficult, especially in the school context, to establish disciplinary meaning-making as a shared line of historical reasoning in classroom discourse (see Popa, 2022). The influence of the abovementioned presuppositions is one reason students’ meaning-making processes often lack substantiation based on historical evidence (Knudsen, 2020; Lee & Chapman, 2015).

One way to change student convictions, according to van Drie and van Boxtel (2008), is to provide history lessons that offer students the opportunity to “reason in interaction with peers, the teacher, and the materials or curriculum used” (p. 160) and thus to “enter a disciplinary community of practice” (van Boxtel & van Drie, 2018, p. 160). *Historical reasoning* is one of the established constructs in the field of history education, and it defines “both goals of history education and the activities that students should engage in to learn history” (van Boxtel & van Drie, 2018, p. 149). The term “activities,” as used by the authors, refers to explicit acts of speech and writing that render students’ engagement with historical phenomena or actions visible. The ability to reason about historical events and phenomena helps students develop a nuanced historical understanding and acquire historical knowledge (historical facts, concepts, and chronology), which is crucial in interpreting and categorizing new information (van Boxtel & van Drie, 2018). Historical reasoning, according to van Drie and van Boxtel (2018), comprises a set of practices, such as asking historical questions, analyzing evidence, using meta-concepts to connect information, and applying substantive concepts to discuss and contextualize historical events.

To enhance historical reasoning, everyday historical narratives should be incorporated into history classes, giving students the opportunity to place them in a broader and more relevant context (van Boxtel & van Drie, 2018). However, due to the complexity of historical meaning-making, knowledge asymmetry becomes problematic, and everyday convictions about historical events are challenging to enrich (Lee & Chapman, 2015). As a result, “subject-related meaning-making is mainly expressed by the history teacher rather than the students” (Knudsen, 2020, p. 37). Therefore, Popa (2022) argues for history education that views students as engaged and reflective thinkers, allowing them to examine historical sources; produce and contest historical knowledge; and understand the interconnectedness of history, the world, and themselves. She proposes a meaning-centered approach that aims “to promote a conscious engagement with what is being learned” (p. 29). This approach is combined with a competency-based approach that targets a disciplinary or expert stance. One means of achieving this is dialogic classroom discourse, which allows students to practice historical reasoning (Zimmermann, 2023).

### Challenges in the Implementation of Dialogic Classroom Discourse

Goffman’s (1981) notion of a *participation framework* explicates the role participants play in meaning-making in classroom discourse, an interaction situation that is markedly distinct from naturally occurring conversations. In teacher–student interactions, students and teachers are commonly accustomed to the most prevalent pattern in instructional conversations: the IRE (Initiation-Reply-Evaluation, Mehan, 1979) “or the so-called ‘recitation script’ of closed teacher questions, brief recall answers and minimal feedback, which requires children to report someone else’s thinking rather than think for themselves and to be judged on their accuracy or compliance in doing so” (Alexander, 2020, p. 15). The teacher asks the questions, evaluates students’ answers, and has the prerogative to decide whether to follow new content cues that students bring to the discussion. Thus, students are given little opportunity to participate in the negotiation of meaning and creation of knowledge (Parkinson & Whitty, 2022). In such a lecture-like *authoritative discourse* (Bakhtin, 1935), knowledge is not negotiated but, rather, already in place and must be revealed by the students (Macbeth, 2001). Michaels and O’Connor (2015), highlight the complexity of facilitating a productive classroom discussion that deviates from the traditional IRE pattern: “Orchestrating academically productive discussion—that is, discussion that supports robust learning for each student—involves a multidimensional blend of human interaction mediated by language, often about a complex topic, with an ambitious goal of human learning” (p. 335).

The high demands of conducting dialogic classroom discussions are evident in everyday school practice. The TIMSS international video study revealed that teachers had significantly more talk time than students in all surveyed countries (Hiebert et al., 2003). Teachers’ contributions were generally longer, with over 70% of their statements exceeding five words, while students’ responses were mostly brief (1-4 words). Further studies (Seidel & Prenzel, 2006) corroborate the notion that students have a substantially lower share of talk time as compared to teachers. A binational video study on classroom discussion’s role in conceptual learning in mathematics instruction in 38 secondary-school classes (Pauli & Reusser, 2015) found that 48% of teacher questions were simple recall questions. Students primarily gave brief answers and seldom justified their statements. Additionally, high-performing students spoke more frequently than their moderately and low-performing peers (Lipowsky et al., 2007).

### Toward Dialogic Classroom Discourse

In the 1990s, by observing two remarkably talented teachers, O’Connor and Michaels (1996) identified innovative teacher strategies that expanded classroom talk beyond the widespread classroom discourse characterized by the IRE pattern. These teaching moves lead to a classroom discourse characterized by a participant framework (O’Connor & Michaels, 1996) that allows students to contribute as central actors, rather than merely providing keywords (Kobarg & Seidel, 2007). Consequently, behaviors during these teachers’ classroom discourse resembled those of natural conversations. Based on their classroom observations, O’Connor and Michaels (1996) emphasize the importance of participant framing in defining the role of students in the process of meaning-making during teacher–student interactions: “Simply put, the participant framework encompasses the ways that speech event participants are aligned with each other or in opposition to each other, and moreover, how they are positioned relative to topics and importantly, even utterances” (p. 6). This includes learners accepting one another as “sources of knowledge” and co-constructing narrative strands (Zimmermann, 2023).

Thanks to various pedagogical approaches (i.e., *Accountable talk, Dialogic Inquiry*, and *Thinking Together*; for a summary, see Kim & Wilkinson, 2019) developed and tested by researchers and educators, dialogic classroom discussions have been promoted through various TPD programs over the past 30 years (Alexander, 2020; Resnick et al., 2018a). Current research on dialogic classroom discourse indicates differences in teacher–student interaction and student roles as compared to those in IRE talk. These novel patterns of teacher–student interaction have been found in mathematics and science subjects (i.e., Osborne et al., 2019; Scott et al., 2006; van Vondel et al., 2017; Wells & Arauz, 2006) and second-language learning (i.e., Walsh, 2002). Furthermore, various studies have demonstrated an increase in students’ talk share (Chen et al., 2020; Michaels & O’Connor, 2015; Moser et al., 2022; Sedova et al., 2019) and improvements in students’ discursive competencies (Heller & Morek, 2015; van der Veen et al., 2017) due to more dialogic classroom discourse. These changes positively affect subject matter learning (i.e., Ing et al., 2015; Jay et al., 2017; Pauli & Reusser, 2015; Webb et al., 2014), as well as student motivation (Böheim et al., 2021; Kiemer et al., 2015).

### The Promotion of Dialogic Classroom Discourse Through Accountable Talk

Resnick et al. (2018a) point to the potential of classroom discourse “to teach students to think—to *make* knowledge” (p. 14). They call this type of talk “Accountable Talk” (Michaels et al., 2010), in which “the teacher’s goal is to sustain a *teacher-led* but *student-owned* process of shared reasoning that ultimately leads to a more fully developed, evidence-backed conclusion, solution, or explanation” (Resnick et al., 2018b, p. 15). Accountable Talk is employed as a pedagogical strategy to cultivate dialogic discourse within the classroom. The aim of this approach is to establish a dialogic participant framework and support teachers in engaging students as accountable contributors in the co-construction of meaning.

Three accountabilities form the standards of this productive talk. First, in Accountable Talk, students are held *accountable to the community* by actively listening to their peers’ contributions, respecting diverse perspectives, and connecting their statements to previously discussed ideas. Both the teacher and the students function as integral parts of a knowledge-building community. The teacher creates an environment that encourages students to contribute their thoughts and fosters their independence as creators of meaning. Second, students are held *accountable to knowledge* by making evidence explicit, using appropriate resources as evidence, and building on a commonly shared understanding of concepts. Third, students are held *accountable to reasoning* in that they must make validly substantiated claims; provide a clear and comprehensible justification for their response; and combine their own perspectives with opposing interpretations, perspectives, and points of view (i.e., Michaels et al., 2008; Zimmermann, 2023). We can only infer a learning-effective classroom discussion in the context of Accountable Talk if all three quality dimensions are met.

This type of discourse is distinguished by its teacher-led yet student-owned nature (Resnick et al., 2018a) and applicable across various subjects due to its generic character (Pauli & Reusser, 2018). By creating “interactional spaces in which students are positioned as contributors whose inputs are recognized and credited” (Lipponen & Kumpulainen, 2011, p. 813), students become accountable agents in meaning-making processes in classroom discourse. Thus, the roles of all participants differ from those generally observed in whole-class discourse (Hiebert et al., 2003).

Various studies have employed the Accountable Talk approach to assist teachers in fostering dialog-based classroom discussions as part of a TPD (e.g., Moser et al., 2022; Michaels & O’Connor, 2015; van der Veen et al., 2017). In our research project Socrates 2.0, we also based our TPD program on the Accountable Talk approach developed by Resnick et al. (2018a). Building on prior research that demonstrated the positive impact of dialogic classroom discussions on student learning, our prior analysis showed that teacher and student behavior in classroom discussions evolved in a direction consistent with dialogic student-teacher interaction. This included an increase in students’ talk share, a higher number of participating students, and changes in the type of student contributions (see Moser et al., 2022), as well as the development of teachers’ skills regarding leading discussions dialogically, which resulted in an increase in the disciplinary qualities of student contributions in history (see Zimmermann, 2023).

These analyses, upon which this article builds, have primarily focused on frequency analyses of language and performance, similar to most previous studies of whole-classroom discussion. This overlooks that meaning-making is a *multimodal experience* (Twiner et al., 2021) that includes a variety of semiotic modes, such as gestures, gaze, body posture, prosody, grammar, and lexis (Mondada, 2014). Dinkelaker (2020) states that all students affect the lesson through their presence and participation, whether they speak or not. There is a growing body of research, particularly in science and second-language education (cf. Evnitskaya & Jakonen, 2017; Matsumoto, 2019; Williams & Tang, 2020; Xu et al., 2021), that addresses nonlinguistic semiotic modes in classroom interaction. Studies examining the effects of such modes, such as gaze, body positioning, and hand raising, on classroom interaction indicate a remarkable influence, as is evident in the context of turn-taking organization and the attainment of intersubjectivity (cf. Gardner, 2019; Matsumoto, 2019).

Given the complexity of analyzing interactions during whole-class discussions, most research to date tends to focus either on a single semiotic mode or indicators of increased disciplinary knowledge within classroom interactions. Little attention has been paid to the coordination of various semiotic modes and their effects on joint meaning-making. In addition, this paper follows up on insights derived from our prior research (Moser et al., 2022; Zimmermann, 2023), which provided preliminary assumptions about the potential effects of multimodal communication. Furthermore, to date, studies have predominantly focused on examining the individual contributions of students and teachers, rather than the dynamics of their interactions. This paper addresses these gaps by integrating a multi-semiotic approach and a content-bound perspective to analyze the interplay between student and teacher contributions in classroom discourse. Therefore, in this article, we address the following research questions:

- How is historical meaning negotiated in two dialogic classroom discussion sequences?
- How do nonlinguistic semiotic modes (gaze, gesture, and posture) accompany student engagement in the process of meaning-making?

## Data and Method

### Data

The data analyzed in this article are derived from the study Socrates 2.0. In this study, a TPD format was designed with the aim of helping teachers develop their classroom discussions toward a dialogic participant framework (Pauli & Reusser, 2018). The study focused on a TPD program for history and mathematics teachers conducted over one school year. The TPD program began after a pre-test and included 1- to 2-day input sessions and three practice phases. During the input sessions, the teachers learned about dialogic classroom talk through presentations, videos, and discussions. In the practice phases, the teachers implemented the strategies in their teaching, with the support of a “toolbox” containing a brochure of Talk Moves based on Accountable Talk. During each practice phase, every teacher received at least two expert coachings based on videos (Allen et al., 2011; for more details, see Zimmermann, 2023). The present study builds on our previous research (Moser et al., 2022; Zimmermann, 2023) and aims to shed light on student engagement in classroom discourse by examining recorded class discussions at a micro level. In previous quantitative analyses, we identified moments of broad and substantive student participation (Moser et al., 2022; Zimmermann, 2023). In the present article, two classroom discussions containing such student participation, which are therefore recognized as examples of good practice, are examined to gain further insights into the nature of student participation and the instructional work of the teachers by meticulously deconstructing the participation of students and teachers in the meaning-making process.

The selection of good practice examples (sequences of broad and substantive student participation) is based on the following criteria: classroom discussions (1) in a history classroom, (2) after the intervention, (3) with a high level of student participation (>50% talk share; Moser et al., 2022; Zimmermann, 2023), (4) with a high proportion of elaborated student contributions (Moser et al., 2022; Zimmermann, 2023), and (5) with a high proportion of dialogic teacher moves (Moser et al., 2022; Zimmermann, 2023). The data were collected in two classes of students aged 14 to 15 years. These were two classes in rural schools with students from diverse migration backgrounds. The two teachers voluntarily enrolled in this professional development program. The content of the program, which focused on dialogic classroom discussions and Accountable Talk, was new to them (Table 1).

**Table 1.** Case Descriptions.

Description	Class R	Class T
Number of students	15 students	20 students
Student age	14–15	14–15
Teacher experience	25 years	35 years
School level	Low track/ninth	Intermediate track/ninth
Duration of classroom discussion	12 min 43 s	7 min 49 s
Topic of classroom discussion	Change and continuity of social inequalities (past and present)	Credibility of historical sources (contemporary witness, features of different types of film)
Guiding question	Are the causes for the unequal treatment of population groups still the same today?	How do you construct your view of the victims of the Shoah?
Preceding activity	Group work	Group work (placemat method)
Seating arrangement during classroom discussion	Group tables (four students)	Group tables (4–6 students)

**Figure 1.**
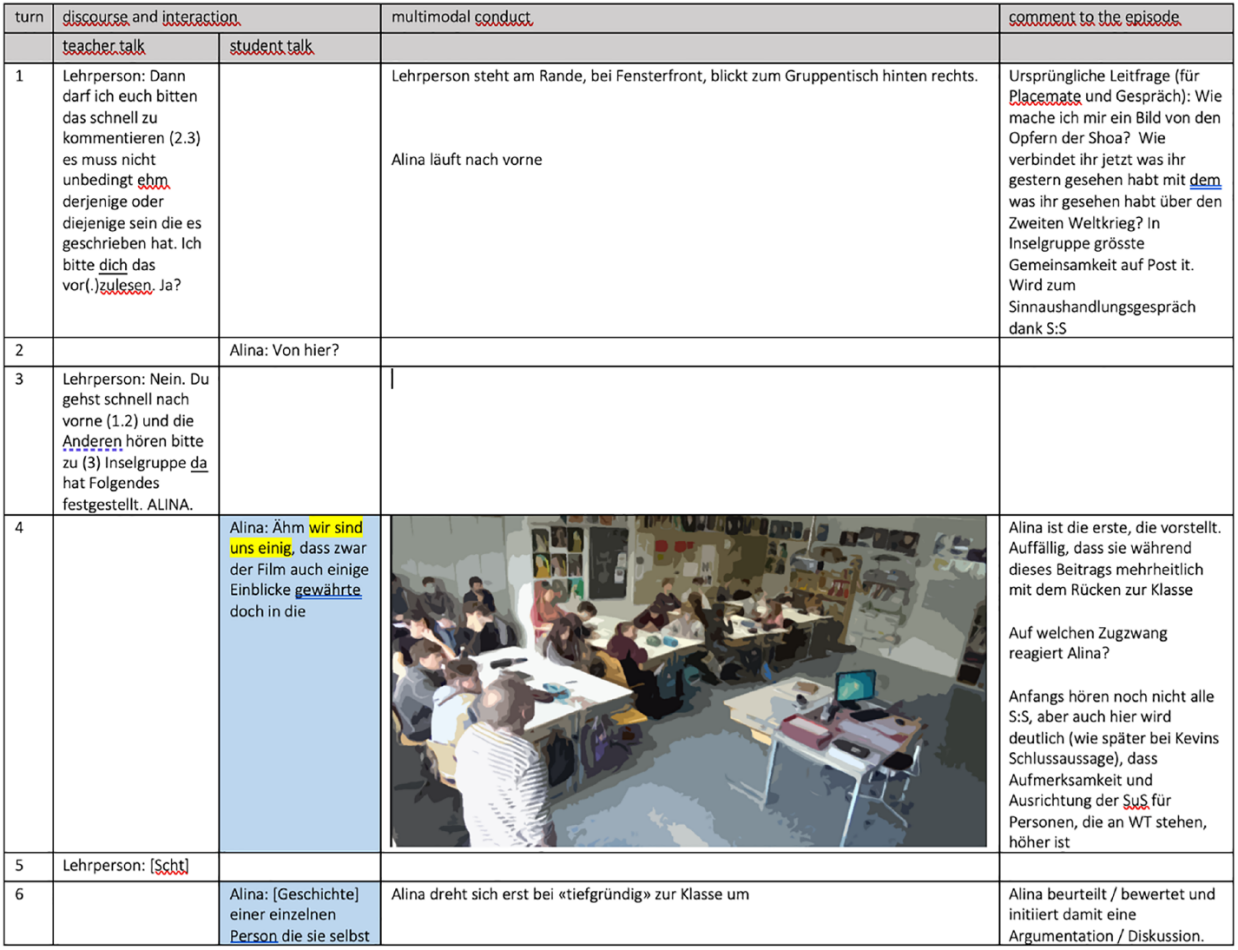
Multimodal protocol.

Video recordings of the whole-class discussions were produced with a two-camera setup (cf. Dinkelaker, 2018; Dinkelaker & Herrle, 2009). One camera was directed toward the teacher, and the other was directed toward the class. The videos were transcribed (see Appendix for notation), and data were analyzed using multimodal transcripts (Figure 1).

Our aim was not to create a model that can be generalized to all schools and pedagogical practices but, rather, to offer a detailed, contextualized view of how teachers and students co-construct meaning in classroom discussions with a dialogic participant framework using different semiotic resources.

### Method

This study involves in-depth case analyses drawing on elements of a reconstructive interactional approach (Brandt & Krummheuer, 2000; Gellert & Krummheuer, 2019) grounded in the theories of symbolic interactionism (Blumer, 1986) and ethnomethodology (Garfinkel, 1967), alongside ethnographic methods (Herrle, 2020). These methodologies aim to reveal the development of and participation in the meaning-making process in two teacher-led whole-class discussions during history lessons.

To do justice to the complexity of the multi-semiotic modes of classroom events with regard to the research question, the content-related strands identified as attention centers were analyzed (Dinkelaker & Herrle, 2009). First, the verbal and nonlinguistic strands were independently sequenced and repeatedly reviewed with vision only and sound only from the perspectives of both cameras. Subsequently, the classroom discussions were analyzed with the inclusion of different modes (verbal actions, gaze, gesture, and body posture). Bezemer (2008) states that “by attending to the ensemble of modes used simultaneously, however, a plausible account may be produced of situated interaction” (p. 169). Observations were recorded using a multimodal protocol (Figure 1). Through the integration of sequence analysis, configuration analysis, and constellation analysis, encompassing both simultaneous and consecutive concatenations of actions, our objective was to acquire a more profound comprehension of classroom interaction (Dinkelaker & Herrle, 2009). Due to the limitations of this article regarding length, only the main result of these intertwined analysis methods can be presented using individual examples. For reasons of readability, only the English translations of the transcripts are presented in this article. The original transcripts can be requested from the authors.

## Findings

First, we describe the context of the classroom discussion. Then, we summarize the main findings and present episodes from each of the two whole-class discussions (Class R and Class T) to illustrate specific insights.

### Whole-Class Discussion: Class R

#### Context

**Figure 2.**
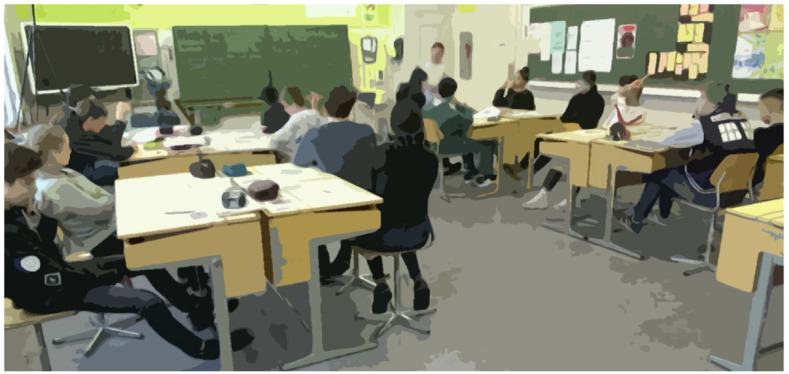
Seating arrangement in Class R.

The selected whole-class discussion is embedded within the double lesson (90 min) of Class R, focusing on the change and continuity of social inequalities in Switzerland (past and present). Students are seated at group tables throughout the double lesson (Figure 2). Prior to this discussion, students work in groups to identify the causes for the exclusion of certain populations based on different historical sources (see in detail Zimmermann, 2023). In the first part of the classroom discussion, the group results are collected on the board. The teacher opens the second part of the discussion analyzed in this article by asking the class the following question: *Do these causes and reasons still have an impact today? Are we all the same, or are some more equal than others?*

#### Main Findings From Class R

The results reveal two phases in this classroom discussion: the initial phase, in which meaning-making is teacher centered, which transitions to a subsequent phase where students take responsibility for themselves. Due to the openness of the central question posed at the beginning of the classroom discourse, students take an active role by introducing racism as the main topic, thereby determining the further content focus of the whole-class discussion. However, initially, the discussion exhibits a rather authoritative character, with the teacher directing the course of meaning-making by querying student contributions and attempting to construct a contradiction (see Table 2, Turn 13). This artificial construction of a contradiction assumes the function of an evaluation, classifying the two student statements. The teacher-centeredness changes when one student *steps in* (Waring, 2011) by evaluating a previous student’s contribution. From then on, the role of the teacher is limited to calling on students who raise their hands. Thus, the teacher not only considers the students’ ideas but also thereafter limits his role to assigning the right to speak. In addition to this shift in orientation from teacher to student, a hierarchy within student authority becomes apparent as (a) only seven of the 15 students engage verbally in the joint meaning-making; (b) not all contributions receive responses; (c) some contributions are verbally built on while others are not; and (d) body language, such as gaze and body alignment, indicates who receives attention and who does not.

**Table 2.** Excerpt 1, Class R: Classroom Discussion Sequence With an Authoritative Character.

Turn	Transcript	Content-related strand
5	Teacher: So, are there still situations today where – where you find that too? Perhaps also in relation to what Mira said earlier, that such – you’ve now spoken of some, or of these minorities (.), that they perhaps feel the way M-Mira described earlier. (2) Lisa?	Emotions of minorities
6	Lisa: So, certainly still with the – so black and white are often still differentiated – so (.) strongly, and I think there are still people who think that black people are worth less (1.5) and there are also still people who (.) still call black people “niggers” and that is – that has never completely stopped, but it’s just become less strong but (.) it hasn’t stopped completely.	Racism, black and white,
7	Teacher: Sam?	
8	Sam: Uhm, with Donald Trump, the Americans, and Mexico (3) that Donald Trump says (.) like the Mexicans (.) are not as developed among us as we are now, now, we don’t want them anymore.	Donald Trump and Mexicans
9	Teacher: So now, earlier – did you hear what Lisa said? She said that there is less black and white today (.) you said that, didn’t you Lisa?	Racism, black and white
10	Lisa: [Yes.]	
11	Teacher: In that direction. And now you bring up an example with America, Mexico – with the Mexicans, where you say they are – can you repeat it? Le-	Donald Trump and Mexicans
12	Sam: [Less developed.]	
13	Teacher: So, less developed (1.8), that’s quite a contradiction, it doesn’t fit together, does it? (1) So, what now, has it become less or not? Well, you just took the example of the black people, you said it’s no longer – it’s become less, it’s not as dramatic as it used to be, and (1.2) uhm, Sam says practically the opposite, just with the Mexicans. (3)	Racism, black and white Donald Trump and Mexicans

#### Participant Framework

The initial episode of the classroom discussion (Table 2) contains the abovementioned contradiction triggered by the teacher. The teacher constructs an artificial contradiction (Turn 13) from Lisa’s contribution, which he does not adequately repeat (Turn 9), and Sam’s initial statements. In contrast to the subsequent phases of this classroom discussion (see also Tables 3 and 4), at this point, the teacher very much controls the course of meaning-making.

**Table 3.** Excerpt 3, Class R: Conclusion of Meaning-Making.

Turn	Transcript	Content-related strand
42	Mira: I think racism will always exist, you can’t (abolish) it like that, regardless of whether it has been abolished by the courts (0.4) the stories are spread and disseminated (.) and told and so on and then (.) the same (value) comes back again (0.7) and then, you consider – you consider black people as inferior again maybe you don’t say it but inside you think the same thing, and sometimes, it also has to do with the (.) um developing country or industrialized country (.) for example, the Africans also got a lot of, uhm, gold from God, I say (.), I say in their country (.), and they didn’t use it (.) and were also robbed by the others. So, they just couldn’t defend themselves “well.” There are always situations where you can’t defend yourself, so (.) you can’t do much against the racists, but you can do something about it so that they’re not punished with death and so on. Efforts have been made against it, but (.) you really can’t abolish it.	Racism, imperialism, socialization
43	Teacher: What can’t be abolished?	
44	Mira: So, racism, so the separation, so (.) separation that you think black people are worth less and white people are worth more.	Racism, black and white
45	Teacher: So Mira says you can’t abolish racism (3.2). Lisa?	
46	Lisa: So maybe in addition to what Mira says, I think maybe from the stories you hear, for example, if you’re outside in the evening and a group of black men come from the refugee center or something like that (.) you might be more afraid of them than if there’s just a group, so that’s how it is for me -	Racism, black and white
47	Teacher: Mhm (affirmative)	
48	Lisa: [If I walk] home in the dark and then I see a lot of black people standing there (.) I might be more afraid than I do when (.) a group of white men were standing there (0.8) [and] I think that’s just because of the stories (.) because we’ve simply heard that (1.2) and yes, simply because we’ve learned it that way	Racism, black and white
49	Teacher: [Mhm (affirmative)]	
50	Lisa: So to speak (.) and through that: we might think simply yes the blacks are perhaps evil (0.5) and the whites are not, so there are also whites, but you have (.) so I above all, become more quickly scared in general when I stand in front of foreigners, of asylum seekers’ homes [than when] (.) than when I stand in front of Swiss people.	Racism, black and white

**Table 4.** Excerpt 2, Class R: Progression of Meaning-Making.

Turn	Transcript	Content-related strand
22	Pete: The problem today is that many people are still interested, because there are always incidents in America and further and from year-to-year hundreds of black people are j-just shot on the streets by policemen, (.) because the policemen get scared where they actually don’t have to get scared, (0.5) because (0.6) 66% of the people killed (.) didn’t even have dangerous weapons or anything with them where they could have harmed people, (.) but nowadays it’s much more in the media, so there are more prison sentences for police officers who do this.	Police, crime, black life matters
23	Teacher: Prison sentences you say?	
24	Pete: Yes.	
25	Teacher: [Yes.]	
26	Pete: Or court cases.	
27	Teacher: Uhm Amir, are you responding to Pete?	
28	Amir: Uhm mm mm (negating) (3) so it’s uh what happens about slavery and racism and so on is certainly stated in the media (.) but not always the truth (.) and sometimes not the whole thing (1.8) because uh there are really things that (1.5) uh are true or not true (0. 5) for example, that the Muslims weren’t even allowed to enter or leave the USA (1.9) that was once a topic (1.2) but after 2 days later (.) there was nothing (0.5) they were allowed to enter and leave again (2.5) yes, so (1.5) (that was) not everything that is said there is true (.) or only half of it is told.	Influence of media, Muslims
29	Teacher: Mhm (affirmative) (2). Reto?	
30	Reto: So I want to refer to what Pete said. So if you had shot a dark-skinned man as a policeman before – in the ’60s and you were caught, so someone could have proven it and there would have been a court case, you would have gotten at most (2.3) five or three years in prison for it. (.) Nowadays you would have gotten (0.9) between 10 and 12 but (0.5) that’s just still (.) too little (0.7) in principle (0.9) because (0.5) we’re in the twenty-first century and you can just say that everyone has the right (0.6) simply to be a human being (1.2), and if you (.) the punishment for an offense against another person is less severe for certain people than it is now for another person, so if you shoot someone white, for example, then you have life imprisonment or 30 years in prison (1.4) so that’s (.) actually still a big difference.	Police, crime, Black Lives Matter

Following this initial phase of authoritative discussion (Table 2), the teacher transitions to a role of nonevaluative engagement with student contributions. He employs clarifying questions (Table 3, Turn 43), distributes the right to speak, and reiterates student contributions through revoicing (Table 3, Turn 45). In this context, students assume the responsibility for responding to and evaluating the contributions made by their peers, as exemplified in Table 3 (Turn 46). An evaluation of the preceding arguments, which concurrently serves as a synthesis of the discourse, is presented in the excerpt in Table 3. Toward the end of the discussion, following an extensive collaborative chain of arguments concerning police conduct and criminal justice practices pertaining to blacks and whites, Mira formulates a final self-initiated conclusion (Turn 42) in response to the teacher’s initial question and the contributions made by her classmates. She elevates the discussion to a meta level by attributing the persistence of racism to socialization (Turn 42). Mira explains that the perpetuation of racist actions and dehumanizing depictions can be attributed to historical events, such as imperialism and the exploitation of Africa, as well as the continued narrative surrounding the value of individuals based on their skin color. She argues that even legal measures are insufficient to eradicate these issues. However, the teacher’s closing question (Turn 43) compels Mira to shift her focus from the meta level and limit her insightful statement to the Black-and-white context. Lisa subsequently supports Mira’s meta-level statement by sharing a personal experience, thereby bridging the generalized conclusion and young people’s everyday lives and connecting historical factors to her own behavior (Table 3, Turns 46, 48, and 50).

#### Roles in the Progression of Meaning-Making

**Figure 3.**
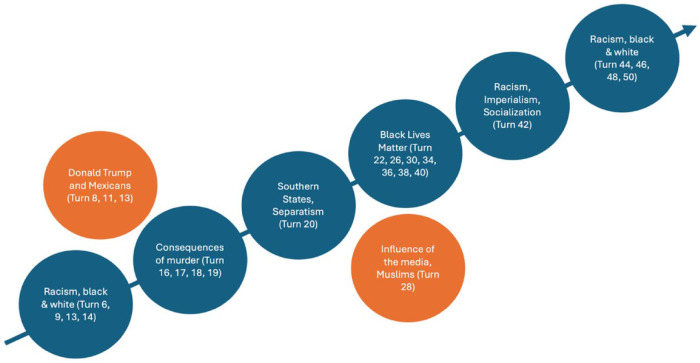
Progression of meaning-making in Class R (Turns 14–21 are not included in the transcripts selected in this article).

Following the initial instance of authoritative discourse (Table 2), the students assume a more proactive role in the process of meaning-making during the subsequent phase (Table 4), whereas the teacher’s involvement is reduced to primarily distributing the right to speak (Table 4, Turns 27 and 29). The students establish a shared line of reasoning by constructing temporal relations (continuity and change) concerning the black-and-white debate initiated by one student and built on by several others. During classroom discussion, two strands of content are not addressed by the teacher or other students and, thus, not included in the joint meaning-making (Figure 3).

The excerpt in Table 4 exemplifies one aspect of the students’ evolving role within the whole-class discussion: the ability to shape the content of the classroom discourse. Pete takes the main floor and expands on previous statements on the “black and white” theme by introducing the role of the police as a new element. The teacher responds to Pete’s statement by asking a brief clarifying question and indirectly prompting Amir to respond to Pete’s contribution (Turn 27). Amir again follows up on Pete’s first statement and the media as a factor, opening up a new content-related strand by addressing the role of the media regarding Muslims (Turn 28). Subsequently, the teacher acknowledges Amir’s contribution with a nod and assigns the class the task of responding to it, refraining from further reacting to or evaluating Amir’s contribution.

Reto (Turn 30) skips Amir’s contribution (Turn 28) and follows up on Pete’s previous statement (Turn 22) by comparing the legal punishment for police officers in the 1960s with that in the 21st century. Amir, who offers a new strand within the meaning-making, is not referred to later, which means that his new content (Muslims) is not included in the knowledge construction of the class. This indicates that not all student contributions are built upon.

#### Accountabilities

In the classroom discussion of Class R, the teacher often responds with moves that promote the *accountability of the community* (“Patric, do you want to respond?” “What do you say to Milo?” “Amir, are you responding to Pete?”). Students establish a shared line of reasoning by building on previous contributions (e.g., Table 4, Turn 30). At the same time, students are *accountable to reasoning* because they justify and explain their points using examples (i.e., Table 4, Turn 22). Nevertheless, students’ contributions lack validly substantiated claims (*accountability to knowledge*), which is illustrated by the following episode (Table 4): all three students participating in this talk sequence express various claims (i.e., in Turn 22, “66% of people killed do not carry weapons”) without *providing warranted evidence* (Michaels et al., 2008). These students attempt to identify and justify continuities of racism, particularly in relation to police actions, but do so without reference to sources. Neither their classmates nor the teacher mention this lack of accountability to knowledge.

#### Amplifying Multimodal Aspects

**Figure 4.**
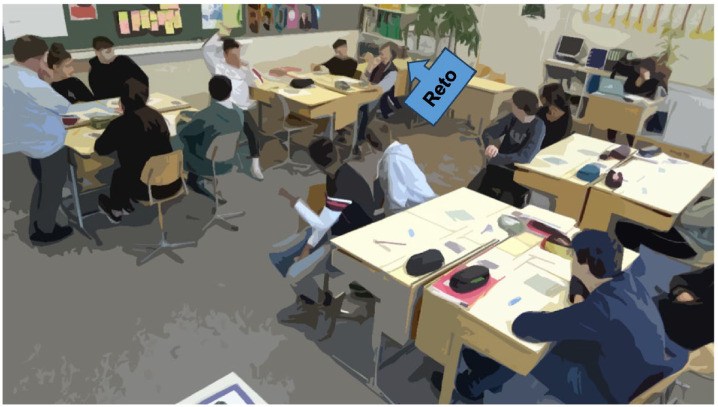
Positioning and gazes while Reto is speaking (Transcript Table 4, Turn 30).

**Figure 5.**
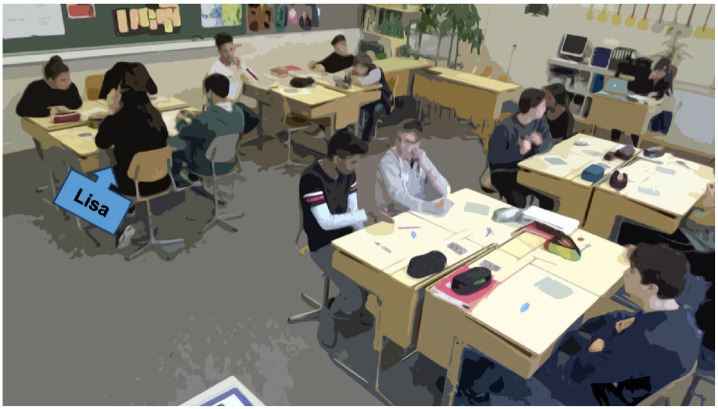
Positioning and gazes while Lisa is speaking (Transcript Table 2, Turn 6).

The interpretation that not all contributions are integrated into the meaning-making process of the class (see Figure 3) is corroborated by nonlinguistic semiotic cues manifested by the participants in this classroom discussion. When granted the opportunity to speak by the teacher, Reto receives noticeably stronger nonlinguistic signs of attention from his classmates, such as gazes and body postures, than other students who also participate verbally. This phenomenon is illustrated in the provided stills (Figures 4 and 5), in which it is evident that when Reto speaks, a higher number of students direct their gaze and adjust their posture toward him (Figure 4) as compared to Amir and Pete during their respective speaking times. Based on the students’ gaze and posture, it can be seen that Reto has established himself as a potential center of attention. A similar pattern emerges when we examine the gaze directions and orientations of students while Mira and Lisa (Figure 5) are speaking. Both girls receive significantly less observable attention from their peers. Another noteworthy observation in the context of the meaning-making process is the absence of any visual aids or visualizations employed to capture ideas or contributions during classroom discussion.

### Whole-Class Discussion: Class T

#### Context

**Figure 6.**
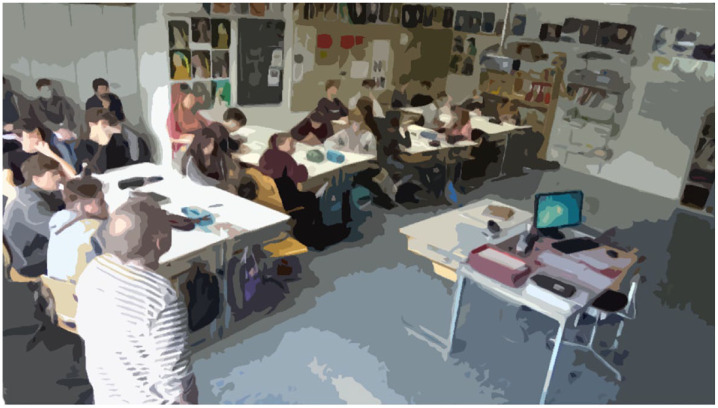
Seating arrangement in Class T.

Class T, the double lesson (90 min) in which the selected whole-class discussion is embedded, addresses the credibility of documents in the context of the Shoah. Students are seated at group tables throughout the double lesson (Figure 6). In the group work (placemat method) preceding the class discussion, the students discuss the following question: *How do you construct your image of the victims of the Shoah?* Beforehand, they were shown the video trailer of the film *Schindler’s List* and a short video excerpt of a contemporary witness (see Zimmermann, 2023). One representative from each group was told to present the most important group results on the blackboard.

#### Main Findings From Class T

In Class T, the initiation of the argumentative nature of the classroom discussion does not stem from the teacher’s central question but, rather, from a student’s presentation of the content of the previous group work. The credibility and comprehensibility of two distinct pieces of evidence are assessed. At this juncture, another student proactively assumes a contrasting stance regarding the assessment of the source’s credibility. Subsequently, the teacher takes on the role of facilitating equal participation by distributing the right to speak and intervening solely to ensure active listening among the students. The teacher allows the students to lead the entire classroom conversation, as is emphasized by his positioning at the side of the room. The students construct meaning by continually introducing new arguments to the discourse, countering, and consolidating the contributions of their peers. Their roles entail embracing and expanding upon previous contributions, explicitly referencing one another by using names, content, and language, including the adoption of specific word choices. As the students in Class T respond to their classmates’ contributions and build upon them, the discourse is characterized by its collaborative and cohesive nature, which is facilitated by both the teacher’s and the students’ active engagement. However, only 10 of the 20 students participate in meaning-making.

#### Participant Framework

In the classroom discourse of Class T, the process of constructing shared meaning is initiated by Mona, who offers a counterargument (Table 5, Turns 9 and 11) to Alina’s statement (Turns 4 and 6) while Kim is in the process of approaching the front of the classroom to present his group’s discussion points. Subsequently, the students respond to and evaluate the preceding contributions. The teacher refrains from posing leading questions, abstains from evaluating statements, and intervenes solely to facilitate active listening among the students (i.e., Turn 10). The teacher’s most guiding action is inviting Mona back into the conversation, giving her the opportunity to respond to her classmates’ reactions (Turn 20).

**Table 5.** Excerpt 5, Class T: Progression of Meaning-Making.

Turn	Transcript (linguistic mode)	Content-related strand
4	Alina: Uhm, we agree that although the film also gave us some insights into the	
5	Teacher: [Sht!]	
6	Alina: The story of a single person, which she tells herself, is nevertheless a (0.8) but very uhm profound and gives a better insight.	Contemporary witness is more credible
7	Kim: I completely agree with Alina. And we also noted something similar, I think.	
8	Teacher: Okay. Wa-wait a minute Kim. Mona?	
9	Mona: But I also think a film can reflect something very well because the people who-	Advantages of the source film
10	Teacher: [Shhhhh!]	
11	Mona: Who made [this] film really dealt with it and tried to recreate this (experience) and that’s why you have a lot of impressions, even though it’s of course different when someone tells you something, but (.) in the film you also see the pictures that you might not have imagined.	Advantages of the source film: visualization, images
12	Teacher: (nods and gives Alina the right to speak by using a hand gesture).	
13	Alina: Well, I don’t think I have to agree with Mona because I think that if someone tells it individually and it really happened that way and in the film, as she said, a lot of information comes together from many different people and whether it was really like that is more of a generalization and when I hear the fate of a real person who tells me the story, I think I can imagine it much better, it’s like a book.	Authenticity of narratives of a contemporary witness
14	Teacher: Discussion is open. Larissa.	
15	Larissa: Well, I think it also depends on how well the person can tell it because sometimes it can also be difficult to really imagine something as it really was because if, for example, a person who has experienced it then really explains it to someone who wants to make a movie, uhm, how it was, and then this person can also affirm, yes (0.7), it was something like that, then you can perhaps imagine it better than if you hear a story from someone and can’t really imagine it, then you just have to somehow create something in your head that perhaps wasn’t like that at all.	Quality of the narrative of a contemporary witness: poor quality of storytelling /narratives
16	Teacher: Mhm (affirmative) still about that? Lucy?	
17	Lucy: Uhm, I think that the images from the movie can also be very impressive and (.) also kind of illustrate (.) which (.) if you just tell it, it’s not like that, but I’m not 100% sure if it was exactly like that or if something was added to make the movie more interesting	Advantages of the source film: fictional character of film
18	Teacher: [Mhm (teacher nods)]	
19	Lucy: [which] often happens.	
20	Teacher: How does that look to you (LP looks at Mona)? Well, there’s controversy. (teacher laughs)	

#### Roles in the Progression of Meaning-Making

**Figure 7.**
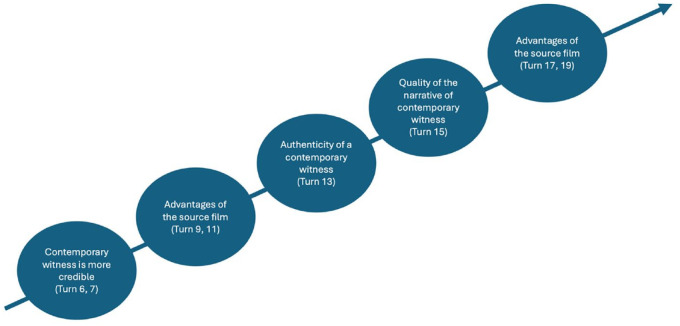
Progression of meaning-making in Class T (Transcript Table 5).

The excerpt in Table 5 exemplifies the teacher’s willingness to cede control over meaning-making to the students, as well as the students’ readiness to embrace this role. Alina begins presenting her group’s opinion regarding the credibility of different historical sources (Turns 4 and 6). Subsequently, the teacher summons the next group to present their results. To give Mona, who has raised her hand, an opportunity to respond to a statement by Alina (Turns 4 and 6), he interrupts Kim, who is making his way to the whiteboard to present his group’s findings. Mona’s counterargument (Turns 9 and 11) initiates a contentious discussion regarding the merits and drawbacks of two historical sources, namely film and contemporary witnesses. Alina (Turn 13), Larissa (Turn 15), and Lucy (Turn 17) expand on Mona’s contribution by introducing new perspectives that align with the central theme of “evaluating historical sources.” All four girls preface their arguments by explicitly acknowledging the subjectivity of their opinions (“I think,” “I find”), thereby distinguishing them from objective facts. The teacher assumes a limited role in the meaning-making process, consisting of acknowledging the students’ contributions (Turn 18), asking the students to listen (Turns 5 and 10) and react (Turn 14) to one another, and granting them the right to speak (Turns 8, 14, and 16), often employing nonlinguistic means of communicating (Turn 12). Unlike Class R’s conversation, in this classroom discussion, each contribution is built on and developed within the main strand of meaning-making (Figure 7).

#### Accountabilities

Excerpt 1 (Table 5) demonstrates the manifestation of accountability to the community through the actions of both teachers and students. The students collectively pursue a shared line of reasoning and construct their contributions by building on previous statements. This can be observed not only in the content but also in the choice of language used. All the students preface their sentences with phrases such as “I think” or “I find,” emphasizing their individual perspectives. Alina uses the word “really” three times in her statement, and Larissa echoes this usage in response to Alina’s contribution (Turn 15). Moreover, Alina explicitly expresses her intention to engage with Mona’s contribution by stating, “I don’t have to agree with Mona.” This verbal acknowledgment highlights Alina’s commitment to respond to and participate in the ongoing discussion. Toward the end of the whole-class discussion, a student follows the preceding speakers by explicitly referring to them by name at the beginning of his statement (“Well, I can only agree with Miro, Sandy, Lucy, and Alina, and I think . . .”). In terms of the teacher’s accountability to the community, he demonstrates it by providing Mona with the opportunity to respond (Turn 8) to the arguments generated by her initial statements (Turns 9 and 11). The extensive contributions of the learners, which are characterized by their uninterrupted nature, both in terms of their classmates (an absence of restlessness) and the teacher, are noteworthy.

As in Class R, the students in Class T are accountable for their reasoning as they explain and justify their arguments (i.e., Table 5, Turn 11: “. . . because the people who made the film were very concerned with this and tried to recreate this experience. . .”). Accountability to knowledge is not prioritized in this discourse, as students are mainly asked to formulate their personal judgments.

As in Class R, toward the end of the discussion, a student presents a final conclusion regarding the relative credibility of different historical sources (“Yeah, well—I think a combination wouldn’t be bad”). However, ultimately, the different types of knowledge sources are not critically evaluated, as an academic stance would require.

#### Amplifying Multimodal Aspects

**Figure 8.**
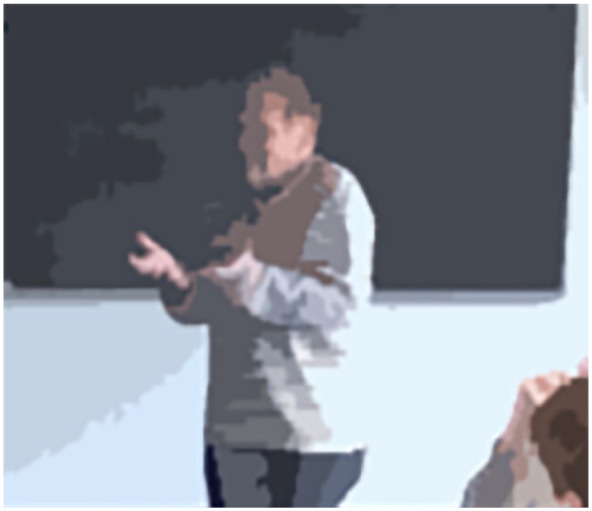
Hand gesture position (Transcript Table 5, Turn 20).

The teacher in Class T communicates strongly through his gestures. One of his typical hand positions (Figure 8) symbolizes an invitation to the students to weigh their arguments without taking a personal stance. The teacher defers the act of taking a position to the class.

**Figure 9.**
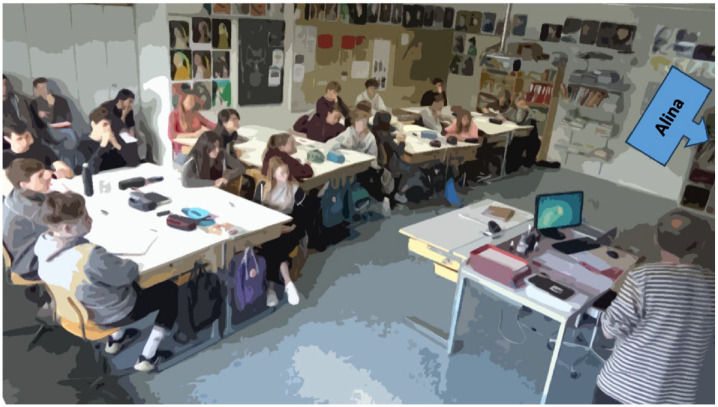
Positioning and gazes of students while Alina is standing in front and speaking (Transcript Table 5, Turns 4 and 6).

**Figure 10.**
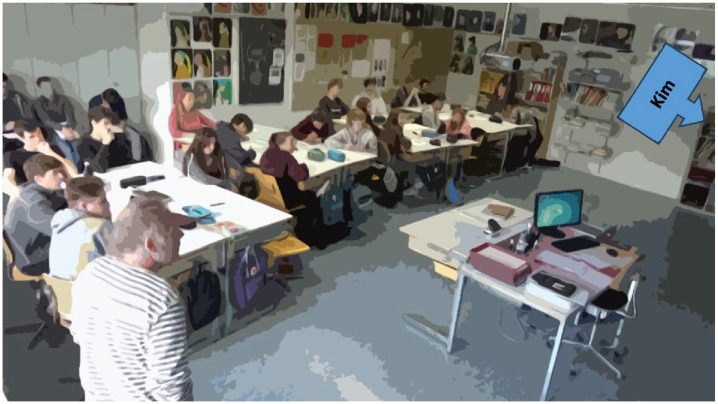
Positioning and gazes of students while Kim is standing in front and speaking (Turn is not included in the transcripts selected in this article).

**Figure 11.**
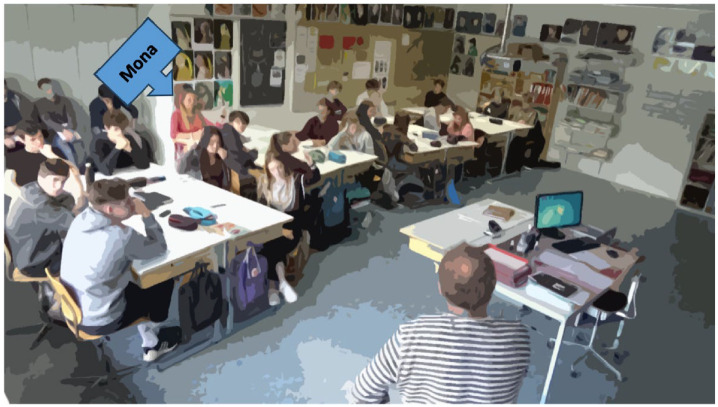
Positioning and gazes of students while Mona is speaking (Transcript Table 5, Turns 9 and 11).

In this class discussion, the interaction between spatial positioning and gaze is striking. As shown in the stills (Figures 9 and 10), Alina and Kim receive significantly more gazes while standing in front than the verbally involved students at the group tables (i.e., Figure 11). They profit from the *concerted attention to the front* (Macbeth, 1992). Thus, in contrast to the situation in Class R, in which a student receives increased attention from peers irrespective of their seating position, Class T demonstrates that spatial positioning influences both gaze and body posture.

## Discussion

The micro-analysis presented above sought to unpack the construction of meaning in teacher–student interactions in dialogic history classroom discourse. While previous studies on classroom discourse have concentrated on the frequency analysis of language and performance, this paper highlights the emergence of meaning in teacher–student interaction at a micro level. It focuses on an in-depth analysis of the negotiation of meaning and the interplay between multi-semiotic communication and verbal participation. For this purpose, we investigated two classroom discussions that were identified as dialogic whole-class discussions through prior quantitative analysis.

The main findings indicate that students assume new roles in whole-class discussions, moving beyond the mere provision of keywords, as observed in classroom discussions characterized by prevalent teacher dominance in terms of the construction of meaning (e.g., Kobarg & Seidel, 2007; Pauli, 2006; Resnick et al., 2018a; Walsh, 2006). This new student agency results in the participating students gaining (and the teachers leaving) the *main floor* (Macbeth, 1992), thereby determining the direction of the knowledge construction by deciding which content-related strands should be included and developed and which should not. However, the findings indicate that reducing the teacher–student asymmetry may inadvertently introduce *new imbalances in classroom dynamics*. As students themselves assess the value of each contribution, they determine which contributions to build upon and which to disregard, thus hindering other students’ *equitable access to participation* (O’Connor & Michaels, 2019). This *empowerment of students* may result in inequities, as not all contributions are acknowledged in a manner that aligns with *an inclusive pedagogical strategy* (Walshaw & Anthony, 2008). In this regard, differences are apparent between the two classroom discussions. In contrast to Class R, in which not all contributions to the process of meaning-making matter, all verbally participating students in Class T engage in a continuous construction of meaning by consistently introducing new arguments, as well as countering and condensing the contributions of others, thereby making significant contributions to the co-construction of meaning. As building on and countering other statements involve highly cognitively demanding thinking processes, the difference in track (low vs. intermediate) may be a contributing factor.

While the dimension of accountability to the community can be considered present to a satisfactory extent in both classroom discussions, both classes lack a reasonable degree of subject-specific content quality (*accountability to knowledge*). The quality of the accountability to knowledge seems to depend on the interplay between the teacher’s prompts during the discussion and the guiding questions and preparatory tasks. With both teachers refraining from evaluating student contributions and classmates not stepping into this evaluative role, students are rarely prompted to support their arguments with appropriate sources and concepts. Furthermore, the distinct guiding questions and preparatory tasks of the two classroom discussions result in different emphases on accountability to knowledge: students in Class T are prompted to articulate their viewpoints without referring to substantive concepts, whereas those in Class R are encouraged to base their arguments on sources discussed during prior group work. According to Michaels et al. (2008), accountability to knowledge is the most complex and difficult of the three accountabilities, as it “is based explicitly on facts, written texts or other publicly accessible information that all individuals can access” (p. 289). This requires the teacher to provide authoritative knowledge and thus uncover misunderstandings and misconceptions, guiding the class toward the acquisition of accurate concepts (Michaels et al., 2008). As Engle and Conant (2002) state, “By failing to take disciplinary norms into account, students may be less likely to become deeply engaged with disciplinary ideas” (p. 409). In both sequences analyzed, the teachers face the challenge of transferring the responsibility for meaning-making to students while also ensuring that they maintain an adequate level of disciplinary thinking. This observation is even more problematic in the contest of history classes because when fostering historical reasoning, students’ use of historical evidence to support claims constitutes a central skill (van Drie & van Boxtel, 2008). Failing to substantiate contributions or refraining to do so according to disciplinary principles may ultimately result in strengthened subjective presuppositions, as opposed to more nuanced historical reasoning.

Ultimately, the analysis incorporating *nonlinguistic semiotic modes* reveals that in addition to the teacher, students can also be recognized as collective attention centers. The students’ nonverbal communication reflects the disparate valuation of the contributions made by some students. In Class R, gaze and body posture reveal a *hierarchy within the class* that determines who should be listened to and whose remarks should be built on. Given the developmental psychology insight that adolescents are more influenced by peers than adults, both verbal and nonverbal reactions to their contributions can significantly impact their future engagement in classroom discussions. Regarding Class T, the findings highlight the impact of the spatial positioning of the speaking student. Furthermore, the use of nonlinguistic semiotic modes, mainly hand gestures, and, consequently, the reduction of the teacher’s own speech share are particularly conspicuous in the classroom discourse of Class T. Given the high level of participation observed in this class, the teacher was able to provide *minimal guidance and relied primarily on nonverbal communication* to distribute speaking rights among the students. This observation corroborates the findings of prior studies, which posit that an increased number of talk moves being employed by a teacher in a discussion does not necessarily indicate a more productive or student-oriented classroom discourse (Zimmermann, 2023; Reznitskaya & Wilkinson, 2021). Our analyses indicate that in classes accustomed to active and accountable participation in classroom discourse, teachers can reduce their talk moves, and the control of discourse can be successively transferred to the students.

## Conclusions

The present study adds to a growing body of research highlighting the benefits of dialogic classroom discourse. The findings illustrate the emergence of new student roles and asymmetries due to the altered responsibilities of student-owned classroom talk. Students take on the role of *agentic engaged participants* who determine the course of meaning-making. The question at hand pertains to the role the teacher should assume in order to facilitate the equitable participation of all students. Moreover, the results highlight the inherent tension teachers face as they strive to ensure accountability to the learning community and disciplinary norms. The question arises as to how a student-owned dialogic classroom discussion can avoid devolving into a meaning-market and, instead, foster *meaning-making accountable to disciplinary norms* while actively engaging students. Another challenge lies in synthesizing students’ various lines of thought and steering the discussion toward a substantiated conclusion. Thoughtful reflection on the embedding of the classroom discussion and well-considered questions during the planning phase could provide the teacher with the support needed to meet demands regarding subject matter expertise. Additionally, employing visual tools to illustrate the process of meaning-making could offer valuable support in classroom discussions. Lastly, the question arises as to what disciplinary demands are appropriate for secondary-school students in the context of (oral) meaning-making.

This study’s results suggest that analyzing teacher–student interactions with a focus on both subject content and multi-semiotic elements, as opposed to examining verbal contributions in isolation, allows for a more nuanced understanding of the data. Consequently, future research may benefit from employing a mixed-methods approach to derive more profound insights. Moreover, given that this study highlights the difficulty teachers face in meeting specific subject matter criteria, future research should more thoroughly explore the integration of classroom discourse into coherent lesson designs (see Specjal, 2022; Zimmermann, 2023).

## Appendix A: Summary of Transcription Conventions

(0.0)  Numbers in brackets represent elapsed time measured in tenths of seconds

(.)  Brief pause of less than 0.3 s

,   Temporary fall or rise in intonation

Word  Underlined sounds are louder

[  Indicates beginning of overlapping talk

]  Indicates end of overlapping talk

wo:rd  Colons indicate a stretched sound

(word)  Parenthesized words are dubious hearings or speaker identifications.

(( ))  Double parentheses contain analyst comments or descriptions
